# The influence of medical providers on HPV vaccination among children of Mexican mothers: a comparison between Mexico and the Midwest region of the United States

**DOI:** 10.1186/s12889-019-6718-0

**Published:** 2019-05-06

**Authors:** Mariela Bahena, Marcela Carvajal-Suarez, Amr S. Soliman, Jiangtao Luo, Armando De Alba

**Affiliations:** 1 Department of Health Promotion, University of Nebraska Medical Center, College of Public Health, Omaha, NE USA; 2 Center for Reducing Health Disparities, University of Nebraska Medical Center, College of Public Health, Omaha, NE USA; 3 Department of Community Health and Social Medicine, City University of New York, School of Medicine, New York City, NY USA; 4 EVMS-Sentara Healthcare Analytics and Delivery Science Institute (HADSI), Eastern Virginia Medical School, Norfolk, VA USA; 5 Department of Health Promotion, University of Nebraska Medical Center, College of Public Health, Omaha, NE USA

**Keywords:** HPV, Knowledge, Vaccination, Hispanic/Latinos health, Minorities

## Abstract

**Background:**

Among cervical cancer patients in the U.S., a disproportionate number are Hispanics/Latinos. Also, about a third of patients diagnosed with cervical cancer annually in Mexico die of the disease. Vaccines are available to protect against HPV, the cause of cervical cancer.

**Methods:**

A cross-sectional study was conducted with 200 mothers of Mexican origin in the U.S. Midwest and Xalapa, Veracruz, Mexico. Based on a validated bilingual questionnaire, this study elicited information about knowledge and attitudes regarding HPV vaccination and cervical cancer.

**Results:**

Mothers living in Mexico showed better knowledge about HPV and HPV vaccine (77.8%) than participants living in the U.S. (48%) *p* < .0001. Logistic regression revealed that receiving information about the HPV vaccine from medical providers was a significant predictor of mothers’ willingness to vaccinate their children.

**Conclusions:**

A need for increasing public health education of Mexican mothers in the Midwest on HPV/HPV vaccination, may lead to improving utilization of the vaccination and eventually a reduction of cervical cancer. HPV vaccination for boys is critical for reducing the risk of transmission to sexual partners and decreasing the risk of HPV- related diseases in the population. Therefore, we recommend increasing efforts to vaccine boys and increasing knowledge that boys must also be vaccinated, especially in Mexico.

## Background

Cancer is a major global health problem. As one of the leading causes of mortality, cancer was responsible for an estimated 8.2 million deaths in 2012. Additionally, an estimated 14.1 million new cases were identified worldwide [1].

The Human Papilloma Virus (HPV) is a causative agent of many cancers [2, 3]. The most frequent HPV associated types of cancer include cervical, anal, vaginal, vulvar, penile, and oropharyngeal [4]. Within these cancers, cervical cancer is the most prevalent. More than 90% of cervical cancers are associated with HPV [5]. Among women with cervical cancer in the United States (U.S.), a disproportionate number are of Hispanic/Latino origin. Nationally, Hispanics/Latinos experience about 50% higher cervical cancer incidence rates compared to non-Hispanic Whites [6]. In Mexico, 2017 estimates revealed that about a third of patients diagnosed with cervical cancer annually die of the disease [7]. Clear incidence rates of cervical cancer for Mexico are difficult to find due to scarce statistics on cancer overall. However, the limited data that is available suggests a crude incidence rate of cervical cancer tumors of 8.59 per 100,000 women ages 15 and older in 2012 [8]. The latest rates of new cases of cervical cancer of 2015 in the U.S. among women of undefined ages is slightly lower than Mexico’s at 7.6 per 100,000 [9].

According to the Center for Disease Control and Prevention (CDC), HPV has currently infected about 79 million individuals across the U.S. and annually infects 14 million individuals [10]. In Mexico, it is estimated that at any given time, 5.1% of women are infected with HPV types 16/18, which are the most common types related to cervical cancer among Mexican women [7].

One of the most effective primary prevention efforts in decreasing HPV infection and consequently cervical cancer is HPV vaccination. The HPV vaccine was first introduced in the U.S. in 2006 and in Mexico in 2008. The cancers prevented with the vaccine are cervical (66.2%), anal (79.4%), oropharyngeal (60.2%), vaginal (55.1%), and vulvar (48.6%). HPV vaccines are therefore recommended for all preteens, including boys, and preferably before engaging in sexual activities to protect against infection [11]. In 2012, Mexico’s National Institute of Public Health began including the vaccine in their National Immunization Card for all girls as early as age 9 [12]. For Mexican girls, they could now receive the vaccine as part of their routine course of other childhood vaccinations. The latest immunization card in Mexico, from October 2015, has promoted a three-dose series [13]. In the U.S., the vaccine is currently given in two doses following a change in CDC recommendations [10, 14].

First-dose rates and completion rates for the vaccine vary across the U.S. by racial and ethnic groups [14, 15]. In Nebraska, coverage estimates for two or more HPV doses in 2016 were higher among Hispanic/Latino adolescents (56.1%). However, when compared to non-Hispanic White adolescents (49.2%), these rates continue to be below the rates of the other recommended vaccines and below the Healthy People 2020 vaccination goal of 80% [16]. In contrast, Mexico’s population has consistently had a higher vaccine initiation and completion rate than the U.S. and even higher than the Healthy People 2020 set goal. When first introduced in 2008, first-dose coverage among the targeted girls of ages 12 to 16 in an initial 125 municipalities, was 98%. The three-dose coverage was at 81%. A year later, Mexico expanded its vaccination program to 182 municipalities and to girls between the ages of 9 and 12. At the time their dosing schedule included the third dose. As a result, these numbers saw a slight decrease [17].

Previous studies have documented low levels of awareness and knowledge about HPV, the vaccine, and HPV’s role in the etiology of cervical cancer among Hispanics/Latinos living in the U.S. [18, 19]. Studies in Mexico have found that some of the best indicators for not vaccinating children against HPV were: not knowing about the vaccine and not knowing about STIs [20]. Additionally, the few Mexican studies conducted on the understanding of the vaccine, have found that knowledge of its benefits and the risks of HPV infection strongly correlate with acceptance of the vaccine for teenage daughters among their mothers [21]. Other studies have found that despite low levels of knowledge of the vaccine, there is a high level of willingness to be vaccinated from women and for their daughters. However, it is important to note that the reasons for not accepting the vaccine were based on misinformation about the vaccine [22].

Considering these differences in vaccination rates and the identified knowledge gaps in Hispanic/Latino groups, we developed a two-site parallel study in Xalapa, Veracruz, Mexico and Omaha, Nebraska, U.S. These cities were selected due to their similarities in population size and their relationship as sister cities. We conducted this study to examine the knowledge and beliefs about the HPV vaccine and the potential reasons for the HPV vaccination uptake rates among Mexican nationals. We initiated this study with the intention of generating new insights that may lead to interventions to improve vaccination rates of individuals in the U.S. and Mexico.

## Methods

### Study aim and design

This cross-sectional study was conducted between May and July of 2017 to assess how knowledge about HPV and the HPV vaccine plays a role in a mother’s decision to vaccinate her child. In the U.S., recruitment was made possible through a partnership between the University of Nebraska Medical Center (UNMC), OneWorld Community Health Centers Inc., and the Mexican Consulate at Omaha. In Mexico, recruitment was performed at the Dr. Gaston Melo Health Center through a partnership between UNMC and the University of Anáhuac Xalapa, School of Medicine. In both settings, the interviews were conducted by research members of our team who are native Spanish speakers.

### Setting

In the U.S., our participants were recruited from the waiting room of the Mexican Consulate in Omaha, Nebraska. The Mexican Consulate has a health office called Ventanilla de Salud (VDS) which, in coordination with OneWorld Community Health Centers, Inc. offers health care education and promotion for disease prevention to people who visit the consulate. The VDS facilitated the private space used to interview the participants of the study.

In Mexico, participants were recruited from the Dr. Gaston Melo Health Center, a community health center in Xalapa, Veracruz. Clinicians provided assistance in identifying potential participants by introducing the study in a private medical office at the end of a patient’s appointment which, also served as the private space for the interview.

### Participants

In the U.S., to be eligible for the study, respondents needed to be female, mothers of Mexican origin living in Nebraska or a neighboring state, be at least 19 years of age, have a daughter and/or son between the ages of 9 and 18 living with her. In Mexico, the inclusion criteria were the same, with the exception that the participants had to be of Mexican origin living in Xalapa, Veracruz. In both settings, verbal consent was obtained before beginning each questionnaire.

In the U.S., 132 potential participants were approached and invited to participate in the study. A total of 100 participants agreed and completed the survey.

In Mexico, participants were invited from the family medicine service area of the clinic. A total of 118 individuals were approached to complete the questionnaire. 12 of those individuals refused to participate and six did not complete the questionnaire. The data analysis was conducted on the remaining 100 completed questionnaires. Anonymity was guaranteed at all times.

### Data collection

#### Study questionnaire

A questionnaire was developed in both, English and Spanish, based on validated bilingual instruments used in the U.S., [18, 23–28]. Both versions of the questionnaire had four main sections. These sections included demographic characteristics of the mothers and children, mothers’ experience and satisfaction with received medical care for themselves and their children, and mothers’ knowledge, awareness, beliefs, and experiences dealing with HPV and the HPV vaccine.

Three of the research members of our study who are of Hispanic/Latino origin and native Spanish speakers separately reviewed the Spanish and English versions to assure correct translation and cultural adaptation. After consensus, the questions were modified and adapted.

The study questionnaire used in the U.S. consisted of 76 questions and only differed from the questionnaire used in Mexico in its demographic characteristics section. In the U.S., participants were given the option to complete the English or Spanish version of the questionnaire. Most of the questionnaires were administered in Spanish. In Mexico, the questionnaire consisted of 72 questions due to the adaptation of the demographic questions. The rest of the sections remained the same as the instrument used in the U.S.

### Measurements

### HPV knowledge

HPV knowledge was assessed by affirmation or negation of 16 statements. These statements related to the perception of risk, detection, transmission, prevention, treatment and consequences of HPV infection. The answers to 14 statements were pre-coded as: yes, no, and I do not know, and two questions had multiple choice options (14 items, valid *N* = 197, Cronbach’s alpha = 0.818). One point was assigned for correct answers to the first 15 questions and three points were assigned to the last question for which more than one answer was allowed. We scored the “I do not know” option as zero. The maximum total possible score was 19 points. The level of knowledge was assessed by adding the points. The total score was divided into three categories according to the 75th, 50th and 25th percentile: high (15 or more points), medium (9 to 14 points) and low (8 or fewer points). Due to low frequency in the results for the high level of knowledge, we used two separate categories for level of knowledge: poor (score of 7 points or less) and good (score of 8 points or more).

#### HPV vaccination beliefs

HPV vaccination beliefs were assessed from 12 statements and answers were yes/no/I do not know options (12 items, valid *N* = 198, Cronbach’s alpha = 0.734). Each statement asked about the importance, need, and benefits of vaccinating children in general. In addition, participants were asked about the benefits, safety, and appropriateness of HPV vaccination for children in the age group of 9–12 years. We assessed the beliefs about adverse behavioral consequences of HPV vaccination by using two questions on the Likert scale. We asked if vaccinating their children would send either of these messages: 1, it is okay to have sex and 2, she/he does not have to use safe sex practices.

### Data analysis

Descriptive statistics were used to summarize the data for each research site. We used Pearson chi-square or Fisher exact test to investigate associations between variables of interest. We performed a logistic regression with adjustments for those variables that showed association with the response variable.

A logistic regression model (LRM) was conducted to identify potential predictors of mothers’ willingness to give at least one dose of the HPV vaccine to at least one of their children in the age range 9–18. A stepwise selection model was used. We controlled for country of residence, health insurance status of mothers and if mothers’ received information about the HPV vaccine from their medical provider or not as well as their HPV knowledge. Because of the statistical significance level of the LRM, we conducted statistical tests for the items that assessed the knowledge and beliefs to see if independent variables were associated or different between these two groups.

Two-sided tests were used for testing all the hypotheses. A *p*-value less than 0.05 was considered statistically significant. For the statistical analysis, SPSS version 23.0 was used.

This research was approved by the UNMC Institutional Review Board (IRB) and by the Academic Research Review Committee of the University of Anáhuac Xalapa.

## Results

### Socio-demographic characteristics

A total of 200 participants were recruited (100 from Mexico and 100 from the U.S.). The mean age of the mothers in the U.S. was 40.76 years (SD 6.2). The mean age of mothers in Mexico was 38.95 years (SD 6.6). Most participants had basic education levels of up to 9 years (64% in the U.S and 72% in Mexico). The majority had a stable life partner and were either married (57% in the U.S and 26% in Mexico) or cohabiting (45% in Mexico and 23% in the U.S) and almost half were dedicated to housework (47.5%). Mothers with at least one eligible child vaccinated against HPV were higher in Mexico (49%) than in the U.S (35%). Characteristics of the study population by country of residence are shown in Table 1.

**Table 1:** Socio-demographic characteristics of mothers by country of residency (*N* = 200, otherwise noted)

Characteristic	U.S.		Mexico		*P*-value
	N(%)	M (SD)	N(%)	M(SD)	
Age of the mother (Years)		40.76(6.2)		38.95(6.6)	
27–37	29(29)		46(46)		0.037
38–48	61(61)		44(44)		
49–59	10(10)		10(10)		
Marital Status					
Married	57(57)		26(26)		<.0001
Single	13(13)		12(12)		
Cohabiting	23(23)		45(45)		
Other	7(7)		17(17)		
Mothers’ education					
Middle school or less	64(64)		72(72)		0.2691^a^
Some High School or High School graduated	21(21)		20(20)		
Some college or technical school or higher	15(15)		8(8)		
Employment Status					
Homemaker	50(50)		45(45)		0.3352^a^
Employed (includes Self-employed)	46(46)		46(46)		
Unemployed (but want to work)	3(3)		8(8)		
Student	1(1)		1(1)		
Health insurance					
No	65(65)		4(4)		<.0001
Yes	35(35)		96(96)		
I don’t know	0(0)		4(4)		
Children’s Vaccination Status					
Daughters with at least one dose of the HPV vaccine (*N* = 131)	21(32.3)		49 (74.2)		<.0001
Sons with at least one dose of the HPV vaccine (*N* = 131)	19(29.2)		1(1.5)		<.0001
Mothers with Children Vaccinated	35(35.0)		49(49.0)		0.045
HPV Knowledge (*N* = 197)					
Poor	51(52)		22(22.2)		<.0001
Good	47(48)		77(77.8)		

^a^No statistically significant at the 0.05 alpha level

### Knowledge of HPV vaccination and beliefs about the HPV vaccine

Overall, the knowledge of HPV and HPV vaccination was good with scores above eight points in 62.9% (*N* = 124) of the participants. However, when analyzing data separately by country, mothers living in Mexico showed better knowledge about HPV and the vaccine (77.8%, <.0001) than participants living in the U.S. (48%, *p* < .0001) (Table 1). The average HPV and HPV vaccination knowledge score obtained from the respondents was 8.02 (range 0–15). We did not find a significant difference for the knowledge level between two countries among mothers who vaccinated their children (*p*-value = 0.7071). However, the knowledge level between the two countries among mothers who did not vaccinate their children showed statistical significance (*p*-value = < 0.0001). Table 2 shows the percentage of correct answers used to assess knowledge of HPV and HPV vaccination by country. The question on prevention of HPV infection contained six answer options in total from which three correct answers were available. Overall, only 16.5% (*N* = 33) of the participants selected the three correct answers: 1. By practicing abstinence, 2. By using condoms and 3. By being vaccinated. Most participants (39.5%, *N* = 79) selected two right answers, 28.5% (*N* = 57) only one right answer and 15.5% (*N* = 31) of the participants indicated that they didn’t know the answer or selected a wrong option.

**Table 2:** Distribution of the correct answers about HPV and HPV vaccine by country of residency (*N* = 200, otherwise noted)

Mothers’ correct answers	U.S. N(%)	Mexico N(%)	*P*-Value
HPV affects not only females	46(46)	47(47)	0.8873
HPV is spread by sexual contact	67(67)	93(93)	<.0001
HPV can cause cervical cancer	62(62)	90(90)	<.0001
HPV is not a rare infection (*N* = 99)	37(37.8)	40(40)	0.7460
There is not a cure for HPV (*N* = 99)	23(23.2)	27(27)	0.5401
HPV can cause genital warts	42(42)	76(76)	<.0001
HPV can cause cancer of the penis	20(20)	(42)42.4	0.0008
HPV does not cause HIV	28(28)	33(33.7)	0.4425
HPV cannot be detected by pap smears	3(3)	7(7)	0.1944
The vaccine is recommended to girls at ages 9–26	52(52)	68(68)	0.0209
The vaccine is recommended to boys at ages 9–26	43(43)	22(22.2)	0.0018
More than one injection is needed for the HPV vaccine	38(38)	52(52)	0.0466
Most medical plans and coupons cover the HPV vaccine	35(35)	43(43)	0.2461
HPV vaccine is expensive	9(9)	22(22)	0.0111
HPV is transmitted by genital skin-to-skin contact	51(51)	74(74)	0.0008
HPV infection can be prevented by practicing abstinence, using condoms and being vaccinated	22(22)	11(11)	0.0361

Most of the women surveyed in Mexico (99%) and the U.S. (94%) believe that vaccines are beneficial. Similarly, 95% of the respondents in Mexico and 94% in the U.S. indicated that vaccinating their children against diseases that can be spread person-to-person is important.

Furthermore, 88% of Mexican and 78% of U.S mothers indicated that if there were a vaccine that prevented cancer, they would want their children to get vaccinated. Mothers living in Mexico were more likely to respond that HPV vaccines are beneficial, safe and appropriate for preteens between 9 and 12 years old than mothers living in the U.S. (84 and 41% respectively). Additionally, they also were more likely to indicate that HPV vaccines are appropriate only for girls (40% vs. 13%) (Table 3).

**Table 3:** Distribution of the HPV and HPV beliefs of Mexican mothers by country of residency (*N*=200, otherwise noted)

Mothers’ beliefs	U.S. N(%)	Mexico N(%)	*P*-Value
Vaccines (in general) are beneficial	94(94)	99(99)	0.118*
Vaccinating their children against diseases that can be spread person-to-person is important	94(94)	95(95)	0.756
If there was a vaccine that prevented the common cold, they would want their children to get vaccinated	78(78)	88(88)	0.059
If there was a vaccine that prevented cancer, they would want their children to get vaccinated (*N* = 199)	97(97)	97(97.8)	1.000*
Vaccines should be required for diseases that can be spread person-to-person	78(78)	87(87)	0.094
HPV vaccines are beneficial	71(71)	95(95)	<.0001
HPV vaccines are appropriate for adolescents (girls and boys as young as ages 9–12)	41(41)	84(84)	0001
HPV vaccines are appropriate only for girls	13(13)	40(40)	<.0001
HPV vaccine is safe	37(37)	60(60)	0.001
Potential side effects of the HPV vaccine would prevent them from getting their children vaccinated (*N* = 199)	21(21)	18(18.2)	0.616
HPV vaccine is difficult to get	2(2)	34(34)	<.0001
HPV vaccine causes health problems	8(8)	14(14)	0.175

*Fisher Exact Test

### HPV vaccination status of children and predictors of mothers’ willingness to vaccinate

The LRM indicated that receiving information regarding the HPV vaccine from medical providers was a significant predictor of mothers’ willingness to vaccinate their adolescents against HPV (Table 4). According to the model, the odds that a mother vaccinated their child against HPV is higher for mothers that obtain information about the vaccine from their medical provider (OR: 8.2 (CI: 1.8–37.7), *p* < 0.01). Additionally, the variable ‘Level of HPV knowledge’ is shown in the model with a borderline statistical significance (Table 5). The level of HPV knowledge might be a potential predictor of mothers vaccinating their children against HPV (OR: 2.0 (CI: 0.9–4.3), *p* = 0.05).

**Table 4:** Logistic Regression Model predicting mothers’ willingness to vaccinate their children against HPV (*N* = 173)

Variable	Odds Ratio	95% C.I. for OR		*P*-value
		Lower	Upper	
Level of HPV Knowledge	2.071	0.985	4.353	0.0548
Medical provider info	8.263	1.808	37.777	0.0065

**Table 5:** Summary of Stepwise Selection

Step	Effect Entered	Score Chi-Square	Pr > ChiSq
1	Medical provider info	15.0437	0.0001
2	Level of HPV Knowledge	3.7629	0.0524

However, the country of residency of mothers was not a significant predictor of mothers’ willingness to vaccinate their children against the HPV. The Hosmer-Lemeshow goodness of fit test indicated that there is not a significant lack of fit as *p* = 0.95 (>.05).

## Discussion

Health is a core element for the well-being of all individuals. While vaccinations have been instrumental in successfully protecting the health of communities globally, differences in knowledge, beliefs, and practices still influence the acceptability of vaccines.

Results from our study report important contrasts. While there was an overall good level of knowledge about HPV and the vaccine among participants, mothers in Mexico showed better knowledge than mothers in the U.S. This is consistent with a previous finding in a study among Hispanic/Latino parents [18].

We found small proportions of participants from both countries recognizing that practicing abstinence, using condoms and being vaccinated are methods for preventing HPV infection. This observation is consistent with results from a mixed-methods study that showed misinformation about HPV and cervical cancer prevention among adolescent of Hispanic/Latino origin [29].

Participants in both countries chose health care professional recommendations as one of the most important factors in their decision to vaccinate their children. This finding is also consistent with previous studies in the U.S. where Hispanics/Latinos consistently cited health care professional recommendations as one of the most important factors in their decision [30]. Additionally, in a recent study, Lechuga and colleagues found a significant association between the intention to vaccinate and health care provider recommendations among Latinas living in the Midwestern U.S. [31]. It is therefore critical to foster physician-patient communication to take action in combating HPV and utilizing HPV vaccines.

This study has its own strengths and limitations. In the U.S., the Mexican Consulate facilitated recruitment due to its high number of visitors. The study minimized bias in the U.S. by using a recruitment site not related to a health care institution which may have been more likely to have access to participants already vaccinated. This allowed the group of obtained participants to be very diverse.

Participants from Mexico were patients from the Dr. Gaston Melo Health Center. However, in our intent to have a representative sample of the community and minimize bias, we selected a community health center which is free of cost and available to all patients regardless of health insurance status or appointment.

Our study was limited by small sample size and the restriction of sampling to the Midwest U.S. and Xalapa, Veracruz. Thus, we cannot generalize the findings from this study to the overall Mexican population in both the U.S. and Mexico. We recognize that there is tremendous variability within the Mexican population across the U.S as there is across Mexico. This means that, as done here, caution must be taken when comparing both groups while recognizing their distinct environment and access to resources.

## Conclusion

Results from the present study suggest a need for enhancing public health education among Mexican mothers in the Midwest on HPV/HPV vaccination and its association with cancer in males and females. Separately, given that mothers in Mexico were more likely to consider the vaccine as only appropriate for girls, it is necessary to use a gender-neutral approach when developing prevention programs in Mexico. HPV vaccination for boys is critical for reducing the risk of transmission to sexual partners and decreasing the risk of HPV-related diseases in the population. Therefore, we recommend increasing efforts to vaccine boys and increasing knowledge that boys must also be vaccinated, especially in Mexico.

Given the importance of prevention efforts, there is a need for more reliable measures to estimate levels of knowledge, awareness and beliefs about HPV and HPV vaccination. These estimates are essential tools for developing educational programs.

U.S. policies should also support vaccination in school settings. In fact, schools in both the U.S. and Mexico should include more health-based education delivered in a culturally and linguistically appropriate manner to parents of Hispanic/Latino origin.

## Abbreviations

CDC: Center for Disease Control and Prevention
HPV: Human Papillomavirus
IRB: Institutional Review Board
LRM: Logistic regression model
SD: Standard Deviation
U.S: United States
UNMC: University of Nebraska Medical Center
VDS: Ventanilla de Salud
